# Draft Genome Sequence of Calcium-Dependent *Novosphingobium* sp. Strain TCA1, Isolated from a Hot Spring Containing a High Concentration of Calcium Ions

**DOI:** 10.1128/MRA.00145-20

**Published:** 2020-04-09

**Authors:** Shun Fujinami, Katsuya Satoh, Issay Narumi, Masahiro Ito

**Affiliations:** a Department of Chemistry, College of Humanities and Sciences, Nihon University, Tokyo, Japan; b Department of Radiation-Applied Biology Research, Takasaki Advanced Radiation Research Institute, Quantum Beam Science Research Directorate, National Institutes for Quantum and Radiological Science and Technology, Takasaki, Gunma, Japan; c Graduate School of Life Sciences, Toyo University, Itakura-machi, Gunma, Japan; d Bio-Nano Electronics Research Centre, Toyo University, Kawagoe, Saitama, Japan; Portland State University

## Abstract

Calcium-dependent *Novosphingobium* sp. strain TCA1 was newly isolated from a water sample from a hot spring containing a high concentration of calcium ions. Here, we report the draft genome sequence of this bacterium, which may be the basis for research on calcium ion homeostasis.

## ANNOUNCEMENT

We report the draft genome sequence of *Novosphingobium* sp. strain TCA1, which shows calcium-dependent growth. We collected a hot spring sample containing a high concentration of calcium from Tsurumaki-onsen in Japan (35.387668 N, 139.277898 E).

The sample was spread on Tris medium agar plates (pH 7.7) containing 5 mM CaCl_2_ and incubated for 2 days at 30°C. Tris medium contained 30 mM Tris base, 7 mM citric acid monohydrate, 0.05% (wt/vol) yeast extract, 50 mM glucose, and 1% (vol/vol) trace elements (1). Dozens of colonies were randomly selected and streaked onto Tris medium agar plates (pH 7.7) with and without 5 mM CaCl_2_. Finally, one strain that did not grow on the Tris medium plate but grew on a Tris medium plate containing 5 mM CaCl_2_ was successfully isolated. Chromosomal DNA was prepared using the UltraClean microbial DNA isolation kit (Mo Bio Laboratories). A partial 16S RNA gene of this bacterium was amplified using primers 27F and 1492R (2, 3) and KOD FX Neo DNA polymerase (Toyobo). The amplified 1,366-bp PCR product was ligated with SmaI-digested pGEM-7zf(+) (Promega) and transformed into Escherichia coli DH5α competent cells. Sequencing of the positive plasmid was performed using universal primers T7 and SP6. This bacterium appeared to be most closely related to Novosphingobium guangzhouense strain SA925, based on partial 16S rRNA gene sequence identity (4), and was named *Novosphingobium* sp. strain TCA1.

Strain TCA1 was grown for 18 h at 30°C in LB containing 5 mM CaCl_2_, and genomic DNA was purified using the DNeasy blood and tissue kit (Qiagen), according to the manufacturer’s instructions.

For sequencing, a library was constructed using the Nextera DNA library preparation kit (Illumina), with an insert length of approximately 300 bp; the library was then subjected to 100-bp paired-end sequencing on the Illumina HiSeq 2500 system (2 × 100 bp). Adapter sequences and low-quality data (Q score, ≥30; read length, ≥10 bases) were trimmed using Trimmomatic v.0.36 (5); 35 million paired-end reads, with an average length of 100 bases, were obtained. The draft genome sequence of *Novosphingobium* sp. strain TCA1 was 6,173,804 bp, with a GC content of 64.3% for the total length, and was composed of 136 large contigs (>500 bp), as assembled by SPAdes v.3.12.0. The *N*_50_ value was 174,423 bp. Automatic annotation was performed using Annotated at DFAST (https://dfast.nig.ac.jp) (6), which predicted a total of 5,649 protein-coding genes. The product names of the predicted protein-coding genes were revised by DFAST. The rRNAs were predicted by Barrnap, which predicted a total of 3 rRNAs, and tRNAs were predicted by ARAGORN (7), which predicted a total of 53 tRNAs. Software analyses were conducted using default parameter settings.

### Data availability.

The GenBank accession number for the draft genome sequence of *Novosphingobium* sp. strain TCA1 is BLJH00000000. Raw sequencing data were deposited in the DDBJ Sequence Read Archive under accession number DRA009695 (GenBank BioProject number PRJDB9061 and BioSample number SAMD00197004).
